# Bevacizumab-induced immunoglobulin A vasculitis with nephritis: A case report

**DOI:** 10.1097/MD.0000000000017870

**Published:** 2019-11-11

**Authors:** Yoko Endo, Kousuke Negishi, Kento Hirayama, Hitoshi Suzuki, Akira Shimizu

**Affiliations:** a Department of Analytic Human Pathology, Nippon Medical School; b Department of Nephrology, Tokyo-Shinagawa Hospital; c Department of Nephrology, Juntendo University Faculty of Medicine, Tokyo, Japan.

**Keywords:** bevacizumab, human galactose-deficient immunoglobulin A1, immunoglobulin A vasculitis with nephritis, onconephrology, thrombotic microangiopathy

## Abstract

**Rationale:**

Bevacizumab—an inhibitor of vascular endothelial growth factor—is effective against various advanced cancers. However, it is associated with the development of hypertension and high-grade proteinuria during thrombotic microangiopathy of the kidney. In addition, there are several reports of immunoglobulin A deposition in the glomeruli, but the etiology is unclear.

**Patient concerns:**

A 67-year-old Japanese man with metastatic rectal cancer underwent low anterior rectal resection, followed by treatment with bevacizumab and SOX (S-1 plus oxaliplatin). Six months later, the patient developed hematuria, nephrotic syndrome, and purpura.

**Diagnoses:**

Renal biopsy revealed endocapillary proliferative glomerulonephritis. Immunofluorescence analyses showed granular mesangial deposition of galactose-deficient immunoglobulin A1. Skin biopsy revealed leukocytoclastic vasculitis.

**Interventions:**

We ceased bevacizumab treatment, while continuing the remaining chemotherapy regimen, as we suspected bevacizumab-induced nephropathy.

**Outcomes:**

Proteinuria and purpura improved immediately after cessation of bevacizumab. We identified this as a case of bevacizumab-induced immunoglobulin A vasculitis with nephritis.

**Lessons:**

To our knowledge, this is the first case of bevacizumab-related immunoglobulin A vasculitis with nephritis, as evidenced by galactose-deficient immunoglobulin A1. When a patient's urine tests are abnormal during bevacizumab treatment, clinicians should consider not only thrombotic microangiopathy but also vasculitis.

## 1 Introduction

Recent advances in onconephrology research and, in particular, studies on the effects of molecularly targeted drugs on the kidney have attracted attention. The molecularly targeted drug bevacizumab, an inhibitor of vascular endothelial growth factor (VEGF), inhibits tumor angiogenesis and is effective against various malignant tumors. Bevacizumab increases the risk of high-grade proteinuria and hypertension.^[1]^ Histologically, most patients show thrombotic microangiopathy (TMA). Bevacizumab decreases VEGF activity levels in the glomerulus, thereby damaging the glomerular endothelium and causing kidney injury.^[1]^

Several cases of immunoglobulin (Ig) A deposition on glomeruli related to bevacizumab have been reported to date,^[1–5]^ but the etiology has not been elucidated. Human galactose-deficient IgA1 (Gd-IgA1) is associated with the pathogenesis of IgA nephropathy (IgAN) and IgA vasculitis with nephritis (IgAVN, Henoch–Schönlein purpura).^[6]^ We report a patient with metastatic rectal cancer treated with bevacizumab who developed hematuria, nephrotic syndrome, and purpura with IgAVN, as was established by Gd-IgA1.

## 2 Case report

A 67-year-old Japanese man underwent low anterior resection of the rectum for T4 N2 M1 stage 4 rectal adenocarcinoma with liver metastasis. The patient had well-controlled hypertension but had a medical history of hyperuricemia. Hematuria was 2+ and proteinuria was 2+ or 3+ in a health examination performed 13 and 12 years earlier (Table 1), but hematuria and proteinuria improved naturally in the last 2 years. Precise urine abnormalities were not evaluated.

**Table 1:**
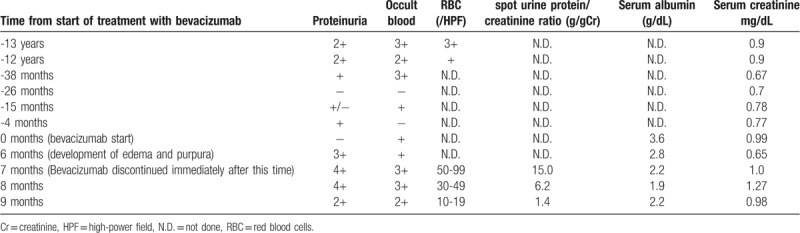
Summary of urine tests and blood tests.

A month after the operation, treatment with bevacizumab and SOX (S-1 plus oxaliplatin) was initiated. After 6 months (total dose of bevacizumab, 660 mg), he developed edema and purpura. After another month, he was referred to a nephrology clinic. He gained 7 kg and developed painful purpuras scattered from the toes of both feet to the distal femurs. There was no prior infection or fever.

Blood pressure was well controlled by nifedipine (40 mg); when it became elevated, the dose of nifedipine was increased to 80 mg, and azosemide (60 mg), furosemide (25 mg), and spironolactone (20 mg) were added to the regimen. The laboratory parameters were as follows. Urinalysis showed a spot urine protein/creatinine ratio of 15.0 g/g creatinine and 50 to 99 red blood cells/high-power field with waxy, fatty, granular, and epithelial casts. Hypoproteinemia and hypoalbuminemia were observed, and serum creatinine was 1.0 mg/dl. Serum IgA was 424 mg/dl (normal: 84–438 mg/dl). Autoantibody and serum complement components were normal.

**Figure 1:**
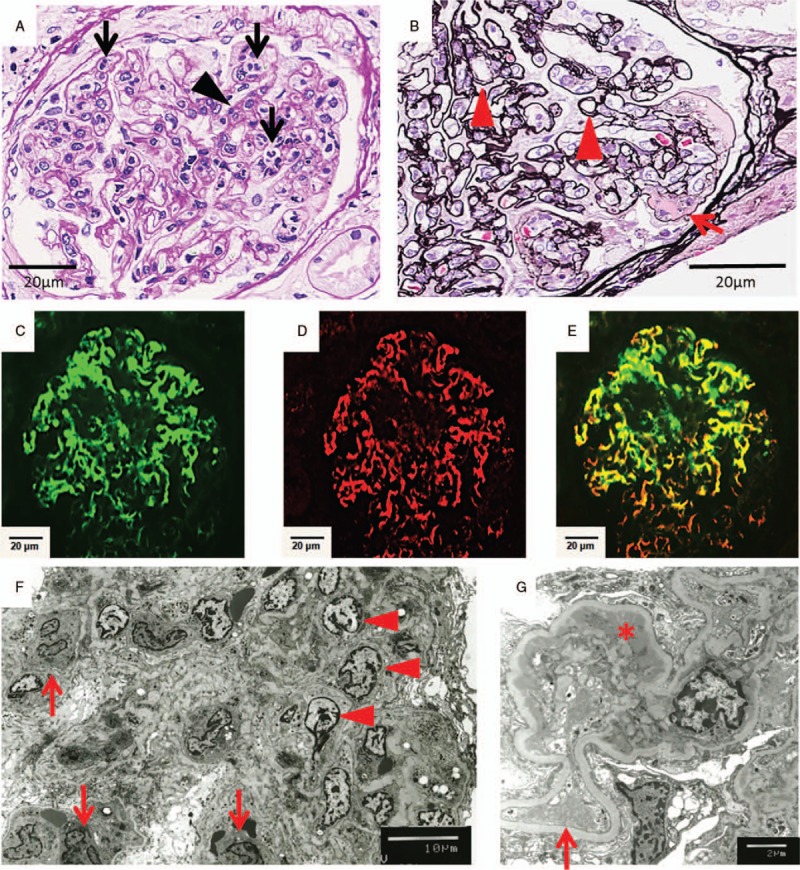
Histological examination of the renal biopsy. (A, B) Light microscopy analysis of the renal biopsy. (A) Periodic acid–Schiff stain: In most glomeruli, many neutrophils infiltrated the capillaries lumen (arrow), and they exhibited endocapillary proliferative glomerulonephritis. Mesangial hypercellularity was mild (arrowhead) (400×). (B) Periodic acid–methenamine–silver stain: Some glomeruli showed double contours of the GBM (arrowhead) and mesangiolysis (arrow) (400×). (C–E) Immunofluorescence analysis showed granular mesangial deposition (200×). (C) IgA, (D) Gd-IgA1, (E) merged image. (F, G) Electron microscopy: (F) Electron microscopy showed mesangial cell proliferation (arrowhead) and inflammatory cell infiltration (arrow) (800×). (G) Electron-dense deposits in the paramesangium (asterisk). Some endothelial cells were enlarged (arrow) (3000×).

Of the 29 glomeruli identified by renal biopsy, 1 was globally sclerotic, and the other 28 glomeruli were enlarged and exhibited endocapillary hypercellularity with neutrophil and lymphocyte infiltration (Fig. 1A). Mesangial hypercellularity was mild. One glomerulus showed cellular crescent. A double contour of the glomerular basement membrane (GBM) was observed in some glomeruli, and 2 glomeruli showed mesangiolysis (Fig. 1B). Tubular atrophic and interstitial fibrotic changes were observed focally; arterial vessels showed mild sclerosis. Immunofluorescence showed granular mesangial deposition of IgA, Gd-IgA1 (Fig. 1C–E), and C3. IgG, IgM, and C1q were negative. Electron microscopy showed electron-dense deposits in the mesangium, with proliferation of mesangial cells and mesangial matrix (Fig. 1F, G). Many inflammatory cells infiltrated the capillaries. Some endothelial cells were enlarged, suggesting damage, but there was no subendothelial edema or thrombosis. There was no foot process effacement, and the epithelial cells exhibited mild swelling.

**Figure 2:**
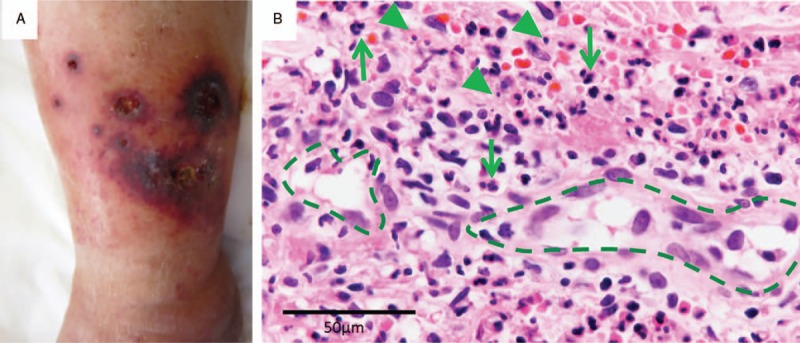
Skin findings. (A) Left ankle with purpuric lesions. (B) Light microscopy of the skin biopsy. Many neutrophils (arrow) infiltrated around vessels (dashed line) with nuclear fragments (arrowhead) (600×).

Leg purpurae were of various sizes and were ulcerated (Fig. 2A). Skin biopsy showed many neutrophils around vessels with nuclear fragments. The findings were compatible with leukocytoclastic vasculitis (Fig. 2B).

We suspected bevacizumab-induced nephropathy and ceased bevacizumab treatment and continued other chemotherapy. Proteinuria and purpura improved immediately (Table 1). The spot urine protein/creatinine ratio improved to 1.4 g/g creatinine; creatinine levels increased to 1.27 mg/dl but improved to 0.98 mg/dl. Blood pressure decreased, and nifedipine was reduced to 40 mg, while azosemide and spironolactone were discontinued.

## 3 Discussion

An active area of research in onconephrology—a new discipline focused on associations between the kidney and tumors—is aimed at determining the effects of molecularly targeted drugs on the kidneys. Kidney-related side effects of bevacizumab include TMA with proteinuria at a frequency of 21% to 64% and hypertension.^[1]^ Podocytes are the main cell type producing VEGF in the glomerulus. VEGF acts on endothelial cells as a paracrine factor and maintains the integrity of the endothelium.^[7]^ Histologically, the characteristic findings of TMA are endothelial cell enlargement, subendothelial edema, mesangiolysis, thrombus, and double contours of the GBM. TMA with bevacizumab frequently shows mesangiolysis and double contours of the GBM.^[8]^ Endothelial cell enlargement, mesangiolysis, and double contours of the GBM were observed in this case, which seem to have been associated with TMA due to bevacizumab.

Only 6 cases of IgA deposition in the kidney caused by bevacizumab have been reported in English or Japanese peer-reviewed journals. Various IgA deposition sites have been reported, such as the subendothelial region, the GBM, or mesangium.^[1–5]^ Yahata et al concluded that IgAN resulted from bevacizumab exposure due to IgA deposition in the paramesangial legion,^[2]^ but the significance of IgA deposition in other cases is not understood well.

Immune complexes containing Gd-IgA1 are important in the pathogenesis of IgAN and IgAVN.^[6]^ Gd-IgA1 is specifically detected in IgAN and IgAVN and not in other renal diseases.^[6]^ Gd-IgA1, anti-Gd-IgA1 antibodies, and soluble Fc alpha receptor form immune complexes and bind to the transferrin receptor of mesangial cells, leading to mesangial cell activation and proliferation. Excess production of Gd-IgA1, immune complexes containing Gd-IgA1, and deposition of these immune complexes in the glomeruli are necessary for the onset and progression of these diseases.^[9]^

IgAN and IgAVN are considered to be related diseases and may develop in the same patient. Apart from extrarenal clinical signs recognized only in IgAVN, nephrotic syndrome is frequently observed in IgAVN. Histologically, vasculitis with leukocyte infiltration is a characteristic feature. Endocapillary and extracapillary inflammation and fibrin deposition are more frequently observed in IgAVN than in IgAN.^[10]^

In this case, the patient developed hematuria, nephrotic syndrome, and purpura, and these improved immediately after the cessation of bevacizumab treatment. Histologically, the main changes in the kidney were endocapillary hypercellularity; mesangial deposition of IgA, Gd-IgA1, and C3; and skin leukocytoclastic vasculitis. Accordingly, we diagnosed this case as IgAVN related to bevacizumab. Light microscopy revealed potential TMA findings, such as double contours of the GBM and mesangiolysis. There was no foot process effacement, and epithelial cell swelling was mild; hence, minimal-change nephrotic syndrome and focal segmental glomerulosclerosis complication were unlikely. TMA as well as IgAVN are considered as the causes of nephrotic syndrome.

Clinically, paraneoplastic syndrome, secondary IgAVN caused by bevacizumab, or the transition of IgAN to IgAVN due to bevacizumab exposure were considered to have potentially caused IgAVN onset in this case. The prevalence of malignant tumors in adult-onset IgAVN is as high as 29% to 43%.^[11,12]^ In this case, the urinary findings were minor before the operation but clearly worsened after 6 months of bevacizumab administration. Thus, paraneoplastic disease was unlikely. Although it is difficult to rule out secondary IgAVN caused by bevacizumab, considering past hematuria and proteinuria, IgAN probably shifted to IgAVN by bevacizumab.

The mechanism by which IgAVN was exacerbated by bevacizumab or how bevacizumab transforms IgAN into IgAVN is unknown. Hyperpermeability of the intestinal mucosa caused by bevacizumab has been reported, indicating excessive IgA secretion and involvement in the onset of IgAN.^[2]^ Gd-IgA1 contributes to the pathogenesis of IgAVN,^[6]^ but its role in causing vasculitis is still under investigation. IgA and anti-epithelial cell antibodies (AECA) bind to the vascular endothelium and promote the migration and activation of neutrophils; this has also been pointed out as one of the causes for the onset of IgAVN.^[13]^ Recent investigations have demonstrated that the concentration of AECA decreases in patients receiving bevacizumab.^[14]^ Moreover, high expression of VEGF was reported in patients with vasculitis and in children with acute-phase IgAV.^[15,16]^ Some reports suggest that bevacizumab administration is unlikely to lead to the development of IgAVN. However, there are reports of bevacizumab inducing the onset of periaortitis and leading to an increase in the rate of thrombosis.^[17,18]^ Vascular disease is caused by an imbalance between vascular injury and epithelial repair. Endothelial progenitor cells are recruited by angiogenic cytokines, especially VEGF, and are essential for epithelial repair.^[19]^ Murakami et al postulated that the inability to mobilize endothelial progenitor cells due to the presence of anti-VEGF antibodies is a risk factor for the development of vascular disease, thereby highlighting the possibility of exacerbating chemotherapy-induced vascular damage.^[17]^

To our knowledge, no cases of mesangial IgA and Gd-IgA1 deposition and skin leukocytoclastic vasculitis caused by bevacizumab have been reported to date; this is the first report of IgAVN related to bevacizumab, as is evidenced by the presence of Gd-IgA1. Proteinuria related to TMA is a common side effect of bevacizumab; however, we think that it is necessary to consider the possibility of bevacizumab-induced vasculitis, such as IgAVN. Thus, this case study may help improve the diagnosis and provide a basis for further research in onconephrology.

## Acknowledgments

We thank Hideaki Murase, and Hideaki Iseki, for introducing such a valuable case to us.

## Author contributions

**Conceptualization:** Yoko Endo, Kousuke Negishi.

**Data curation:** Yoko Endo, Kousuke Negishi, Hitoshi Suzuki, Akira Shimizu.

**Formal analysis:** Yoko Endo.

**Funding acquisition:** Kousuke Negishi.

**Investigation:** Yoko Endo, Kousuke Negishi, Kento Hirayama, Akira Shimizu.

**Methodology:** Yoko Endo, Kousuke Negishi, Hitoshi Suzuki, Akira Shimizu.

**Resources:** Yoko Endo, Kousuke Negishi, Hitoshi Suzuki.

**Supervision:** Akira Shimizu.

**Validation:** Yoko Endo, Kousuke Negishi.

**Visualization:** Yoko Endo, Kousuke Negishi, Hitoshi Suzuki.

**Writing – original draft:** Yoko Endo.

**Writing – review & editing:** Yoko Endo, Kousuke Negishi.

Yoko Endo orcid: 0000-0001-8573-2187.
